# A Strong Anti-Inflammatory Signature Revealed by Liver Transcription Profiling of *Tmprss6*−/− Mice

**DOI:** 10.1371/journal.pone.0069694

**Published:** 2013-07-29

**Authors:** Michela Riba, Marco Rausa, Melissa Sorosina, Davide Cittaro, Jose Manuel Garcia Manteiga, Antonella Nai, Alessia Pagani, Filippo Martinelli-Boneschi, Elia Stupka, Clara Camaschella, Laura Silvestri

**Affiliations:** 1 Center for Translational Genomics and Bioinformatics, San Raffaele Scientific Institute, Milan, Italy; 2 Division of Genetics and Cell Biology, San Raffaele Scientific Institute and Università Vita Salute, Milano, Italy; 3 Department of Neuro-Rehabilitation & INSPE, San Raffaele Scientific Institute, Milan, Italy; National University of Singapore, Singapore

## Abstract

Control of systemic iron homeostasis is interconnected with the inflammatory response through the key iron regulator, the antimicrobial peptide hepcidin. We have previously shown that mice with iron deficiency anemia (IDA)-low hepcidin show a pro-inflammatory response that is blunted in iron deficient-high hepcidin *Tmprss6* KO mice. The transcriptional response associated with chronic hepcidin overexpression due to genetic inactivation of *Tmprss6* is unknown. By using whole genome transcription profiling of the liver and analysis of spleen immune-related genes we identified several functional pathways differentially expressed in *Tmprss6* KO mice, compared to IDA animals and thus irrespective of the iron status. In the effort of defining genes potentially targets of Tmprss6 we analyzed liver gene expression changes according to the genotype and independently of treatment. *Tmprss6* inactivation causes down-regulation of liver pathways connected to immune and inflammatory response as well as spleen genes related to macrophage activation and inflammatory cytokines production. The anti-inflammatory status of *Tmprss6 KO* animals was confirmed by the down-regulation of pathways related to immunity, stress response and intracellular signaling in both liver and spleen after LPS treatment. Opposite to *Tmprss6* KO mice, *Hfe^−/−^* mice are characterized by iron overload with inappropriately low hepcidin levels. Liver expression profiling of *Hfe^−/−^* deficient *versus* iron loaded mice show the opposite expression of some of the genes modulated by the loss of *Tmprss6*. Altogether our results confirm the anti-inflammatory status of *Tmprss6* KO mice and identify new potential target pathways/genes of Tmprss6.

## Introduction

Epidemiological studies suggest that iron modulates the susceptibility to infections/inflammation, but the molecular mechanisms underlying this phenomenon are incompletely understood. The iron/inflammation relationship is reciprocal, since several iron-related molecules (TfR1, Fpn, ferritin, Lcn2, etc.) are transcriptionally modulated by inflammation [1]. Among them the anti-microbial peptide hepcidin, the main regulator of systemic iron homeostasis, is an acute phase protein expressed and secreted by the liver, which provides a critical connection with the immune response [2]. Hepcidin expression in inflammation is activated by IL6 and IL22 [3] through phosphorylated Stat3 (P-Stat3) binding to the hepcidin promoter, in a region closed to the Bone Morphogenetic Protein (BMP) Responsive Elements (BRE) binding sites [4]. Hepcidin binds the sole cellular iron exporter ferroportin triggering its internalization and degradation, reducing iron flux from duodenal enterocytes and macrophages and resulting in hypoferremia, a protective response against microbial growth [5]. Hepcidin-ferroportin interaction in macrophages has been reported to cause JAK2-related transcriptional changes that negatively modulate the cytokine-induced inflammatory response [6], although recently the phosphorylation of JAK2 as a result of hepcidin-ferroportin interaction has been disputed [7].

The type II transmembrane liver serine protease TMPRSS6/matriptase-2 is the main negative regulator of hepcidin, since *in vitro* it cleaves membrane hemojuvelin, the liver-specific BMP-coreceptor in the hepcidin-activating pathway. Genetic inactivation of *Tmprss6* both in mice and human causes severe, atypical iron deficiency, characterized by microcytic anemia and inability to respond to oral iron treatment, because of inappropriately high hepcidin levels [8], [9], [10].

We have previously demonstrated that modulation of hepcidin in mice influences the inflammatory response. The production of pro-inflammatory cytokines is increased upon LPS challenge in iron- and hepcidin-deficient animals and the effect can be abrogated by a short pre-treatment with exogenous hepcidin before LPS injection [11]. In line with this observation, *Tmprss6* KO animals, characterized by chronic iron deficiency with high hepcidin, show a blunted production of inflammatory cytokines and of liver acute phase proteins and reduced tissue macrophages recruitment after LPS, when compared with iron deficient (low hepcidin) mice. These findings suggested that *in vivo* lack of hepcidin and not lack of iron induces a proinflammatory condition, when body iron is low [11]. However, the molecular pathway/s that account for the anti-inflammatory phenotype observed in *Tmprss6* KO mice remain undefined.

The liver plays a crucial role in the response to systemic inflammation, via secretion of acute phase proteins and hepcidin production. For this reason we investigated the whole genome transcriptional profiling of the liver and the expression of selected genes in the spleen in *Tmprss6* KO mice, which are iron deficient with high hepcidin, in comparison with iron deficient (IDA) animals, with low hepcidin levels. The latter approach was performed with the aim of identifying signaling pathway/s activated by chronic hepcidin overexpression and/or *Tmprss6* deficiency, irrespective of the iron status. Here we show that in the absence of *Tmprss6* and in the presence of high hepcidin genes encoding inflammatory molecules are down-regulated, whereas genes connected with the anti-inflammatory response are up-regulated.

## Materials and Methods

### Animals, Diet and Tissue Collections

Mice were maintained in the animal facility of San Raffaele Scientific Institute in accordance with the European Union guidelines. The study was approved by the Institutional Animal Care and Use Committee (IACUC) of San Raffaele Scientific Institute, Milan, Italy. To study the liver gene expression profiling in the absence of *Tmprss6*, we used *Tmprss6* KO mice and iron deficiency anemia (IDA) control littermates, as described by Pagani et al. [11]. Briefly, four weeks old KO male mice, on a mixed 129/Ola × C57BL/6 background [9], were fed an iron-balanced diet (carbonile iron 200 mg/kg, SAFE, Augy, France). Four weeks old IDA animals were maintained on an iron-deficient diet (<3 mg iron/kg; SAFE) for 3 weeks. Inflammation was induced by intra-peritoneal injection of lipopolysaccharide (LPS) (from E.coli O26:B6; 0.1 mg/kg, i.p., Sigma-Aldrich, Sydney, Australia). Animals were sacrificed 6 hours later.

To investigate the role of exogenous hepcidin in the modulation of liver gene expression, seven weeks IDA mice were i.p injected with 100 micrograms/animal of recombinant hepcidin or sterile PBS as vehicle. Mice were sacrificed 8 hours later.

To study liver gene expression modulation by dietary iron, four weeks old male mice were maintained an iron balanced, iron deficient and iron loaded (8.3 g/kg iron, SAFE, Augy, France) diet for 3 weeks. Livers and spleen used for RNA isolation were stored in RNALater (Qiagen, Mississauga, ON, Canada) and processed for quantitative real-time PCR. Livers were also analyzed for total liver iron content (LIC) [11].

### Microarray Analysis

The gene expression profile was determined using the MouseWG-6 v2 Expression BeadChips (Illumina®). On a single BeadChip it is possible to simultaneously profile six samples for more than 45,200 transcripts for each sample. In the first phase of the experiment, cDNA and cRNA synthesis was performed using the Illumina Total Prep RNA Amplification Kit (Ambion), according to the manufacture’s protocol; briefly, 500ng of total RNA, isolated from liver tissue by using the RNeasy Mini Kit (QIAGEN), were reverse transcribed to cDNA with T7 Oligo(dT) Primer, then the double strand cDNA was in vitro transcribed to synthesize cRNA using a biotin-NTP mix. The resulted cRNA was quantified by three replicate measurements using Nanodrop-2000 spectrophotometer and the quality assessed using the Agilent Bioanalyzer. 750 ng of cRNA (150 ng/µl) were then hybridized to the BeadChip at 58°C overnight and the fluorescent signal was developed with streptavidin-Cy3. BeadChips were then imaged using the Illumina® BeadArray Reader, a two-channel 0.8 µm resolution confocal laser scanner and the Illumina® GenomeStudio software. This software was used to elaborate the fluorescence signal to a value, whose intensity corresponds to the quantity of the respective transcript in the original sample. The same software was used to assess the system quality control, including biological specimen, hybridization, signal generation controls and negative controls.

Gene expression data were normalized using the cubic spline algorithm implemented in the Illumina® GenomeStudio software. More than 30,000 genes were investigated with the array experiment, of those a selection of “expressed” genes was done by filtering on the “detection P-Value” parameter. The transcripts whose intensity value was significantly different from that of background (detection P-Value <0.01) in at least one sample of the entire series were considered “expressed” genes in the experiment and on those the following analyses were performed. More than 10,000 genes were appointed as “expressed” in the experiment. PCA (Principal Component Analysis) was done on that group of genes using scripts in Rstudio [12]. LIMMA Bioconductor package [13] was used extract of differentially expressed genes considering a factorial design model and pair-wise comparisons. A post test was used to select putative differentially genes considering genes and comparisons (i.e. “contrasts”) taking into account Benjamini Hochberg multiple comparison correction. The genes passing a cut-off of adjusted P-Value <0.05 and |log_2_ratio| >1 were retained as differentially expressed. The selection of differentially expressed genes considered the following comparison (KO: Tmprss6 KO; IDA: Iron Deficiency Anemia; UT: untreated; LPS: LPS treatment).

KO.UT-IDA.UT.

KO.LPS-IDA.LPS.

IDA.LPS-IDA.UT.

KO.LPS-KO.UT.

(KO.LPS-KO.UT)-(IDA.LPS-IDA.UT) = Interaction.

(KO.LPS+KO.UT)-(IDA.LPS+IDA.UT) = Genotype.

(KO.LPS+IDA.LPS)-(KO.UT+IDA.UT) = Treatment.

A biological term enrichment analysis using Gene Ontology biological process database was performed using DAVID tool [14] [15] considering the background list of “expressed” genes and the single lists deriving from the differentially expressed genes in specific contrasts i.e.: Genotype, Interaction and the single pairwise comparisons in basal and stimulated conditions (KO.UT-IDA.UT, KO.LPS-IDA.LPS).

The enriched categories were selected for being enriched in comparison under investigation and not in the background of “expressed” genes. GoSemSim package [16] was used to calculate semantic distances among Gene ontology biological process terms and the results used to cluster the biological terms so grouping related categories using hierarchical clustering.

The same procedure of enrichment was done considering the KEGG pathway database [17] [18] and the DAVID server [14].

The microarray data were deposited in NCBI’s Gene Expression Omnibus public repository and are accessible through GEO Series accession number GSE46287 [19]. As additional validation of the functional profiling, a repeat analysis was carried out using Gene Set Enrichment Analysis (GSEA) [20] (GSEA Analysis S1).

### qRT-PCR

Two/three micrograms of total RNA were retro-transcribed with the High Capacity cDNA Reverse Transcription Kit (Applied Biosystem), using Random Examers and RNase Inhibitor. Gene expression levels were measure by quantitative real-time PCR using the ABI7900 Real-Time PCR System (Applied Biosystem) using TaqMan Gene Expression Master Mix (Applied Biosystem). Primers used for qRT-PCR are in Table S4. The unpaired 2-tailed Student t test was used to analyze significant changes in gene expression levels (GraphPad Prism Version 5.0a). P-Values <0.05 were considered statistical significant.

### Mouse Immune Array v2.1

RNA isolated from the spleen of IDA and Tmprss6 KO mice, treated or not with LPS, were retro-transcribed as described in the “qRT-PCR” section, pooled and analyzed by TaqMan qRT-PCR using a Mouse Immune Array v2.1 (Applied Biosystem), that allows the evaluation of the expression of about 90 immune-related genes. Hprt1 was used as the housekeeping gene. Unpaired 2-tailed Student t test was used to analyze significant changes in gene expression levels (GraphPad Prism Version 5.0a). P-Values <0.05 were considered statistical significant.

### Western Blot Analysis

Livers were lysed in lysis buffer (200 mM Tris-HCl, pH 8; 1 mM EDTA; 100 mM NaCl; 10% glycerol; 0.5% NP-40) containing a mixture of proteases (Sigma-Aldrich) and phosphatases (Roche) inhibitors. Protein extracts (80 µg) were diluted in Laemmli buffer, boiled 5 minutes, separated onto a 10% SDS-PAGE and then transferred to Hybond C membrane (Amersham Bioscience Europe GmbH) by standard Western blot technique. Blots were blocked with 5% nonfat milk in TBST (0.5 M Tris-HCl, pH 7.4; 0.15 M NaCl and 0.1% Tween 20), incubated overnight with anti-phospho-SMAD1/5/8 (1∶1000; Cell signaling; Millipore), anti-phospho-STAT3 (1∶1000; Cell Signaling), anti-NF-kB p100 (1∶1000; Cell Signaling), anti-NF-kB p65 (1∶200; Santa Cruz Biotechnology Inc.), anti-actin (1∶5000; Sigma-Aldrich). After washing with TBST, blots were incubated 1 hour with relevant HRP-conjugated antisera and developed using a chemoluminescent detection kit (ECL; Amersham Biosciences).

## Results

To study the role of *Tmprss6* in hepatic gene regulation, we performed whole genome expression profiling on individual livers of untreated and LPS-injected Tmprss6 KO vs control IDA mice. Although *Tmprss6* KO mice show a more severe iron deficiency anemia than control mice (Hb 97+/−1.5 vs 126+/−4.4 g/L [11]), liver iron content is comparable in the two groups (106.8+/−14.4 in *Tmprss6* KO mice vs 101.8+/−11.6 µg iron/g liver in IDA animals) and does not significantly change after LPS treatment, as already reported for iron deficient animals [11].

**Figure 1:**
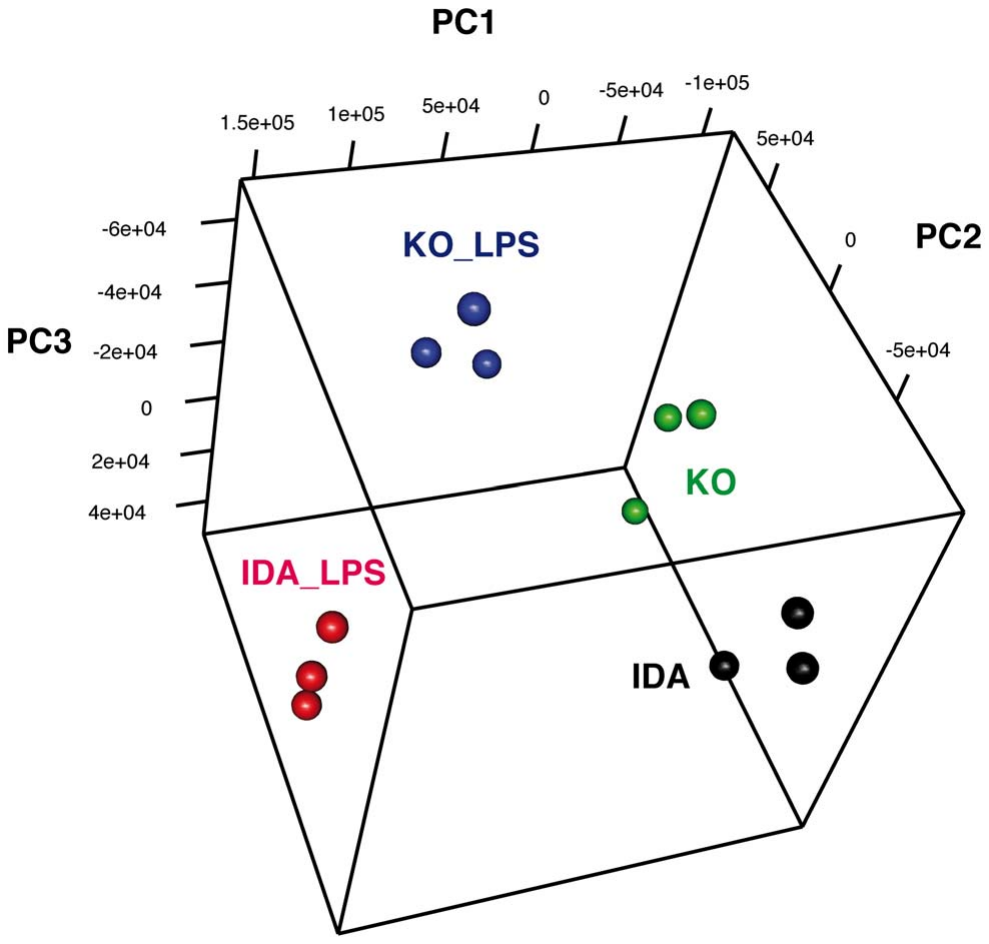
Principal component analysis of microarray data. PCA was made over normalized expression levels of expressed genes in the array (detection P-Value <0.01 in at least one sample). The first 3 principal components accounted for the 85% of explained variance and clustered apart samples coming from the different genotypes and treatments. Black: IDA. Red: IDA+LPS. Green: Tmprss6 KO. Blue: Tmprss6 KO+LPS.

Principal Component Analysis [21] was performed on the subset of expressed genes in the experiment (see materials and methods for working definition of expressed genes). A tridimensional visualization of the first 3 principal components of the various mice groups (**Figure 1**) show that Tmprss6 KO mice formed a distinct group, illustrating the strong impact of Tmprss6 deletion on liver gene expression. A similar situation is maintained also in LPS treated animals, suggesting a different LPS response in the two groups of mice.

### Genes Differentially Regulated in the Absence of *Tmprss6*

To identify genes differentially expressed in the absence of Tmprss6 we used the LIMMA Bioconductor package [13]. The "Genotype contrast” identifies genes differentially expressed in *Tmprss6* KO mice compared to wild-type animals, independently of the LPS treatment. The “Treatment contrast” recognizes genes differentially regulated by the LPS treatment, independently of the genotype, whereas the “Interaction contrast” identifies genes whose expression levels are influenced by both the absence of Tmprss6 and LPS treatment (**Table S1**).

**Figure 2:**
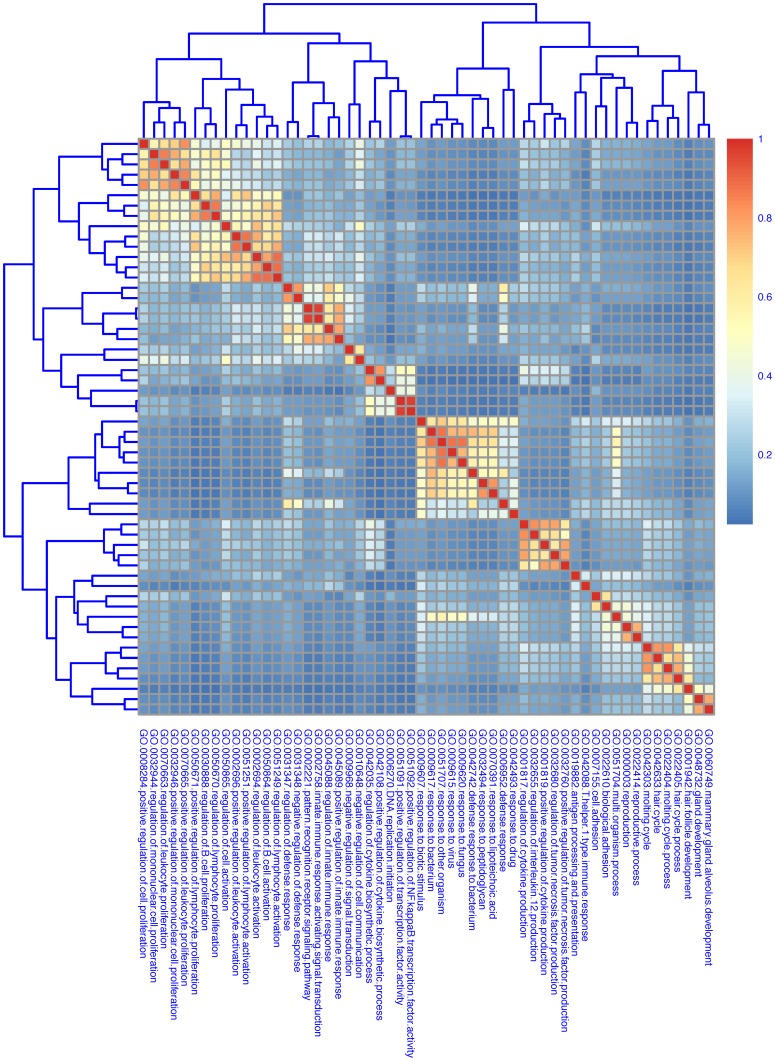
Heat map of clustered biological terms highlighted by differentially expressed genes in the “Genotype contrasts”. The heat map represents semantic similarity among gene ontology (GO) Biological Process (BP) terms. Rows and columns show the list of enriched GO BP terms derived from term enrichment analysis of Genotype significant genes. The colors represent the semantic distances calculated using GOSemSim Bioconductor package. Yellow-red clusters identify groups of terms sharing semantic similarity about biological processes.

**Figure 3:**
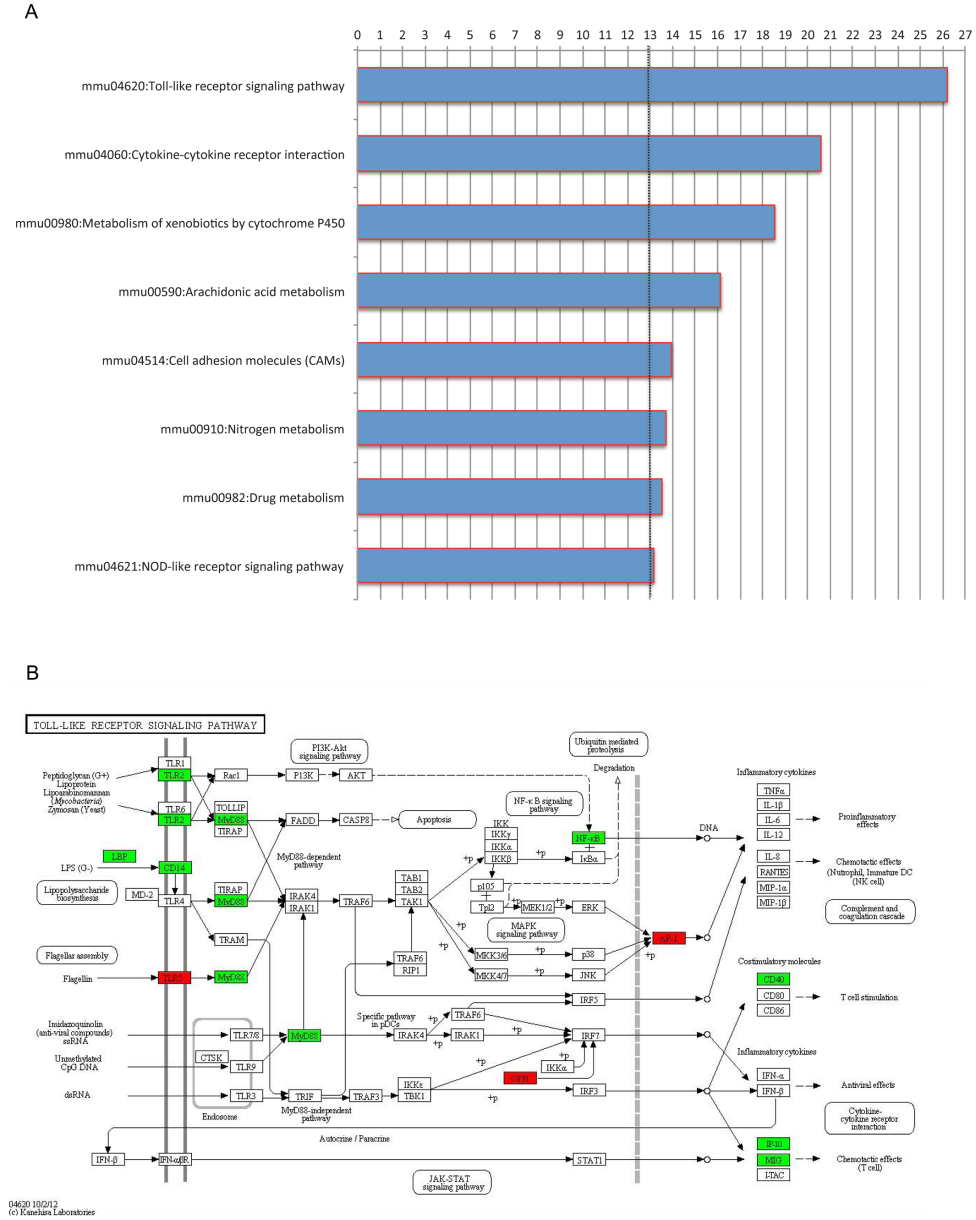
Representations of Kegg pathways enriched in the “Genotype contrast”. **A**) KEGG pathways derived from term enrichment analysis. Bars represent −10*log_10_(P-Value). The dotted line shows significance cut-off at enrichment analysis, which corresponds to a P-Value of 0.05. **B**) Representation of the KEGG Toll-Like Receptor Signaling Pathway, showing up-regulated (red boxes) and down-regulated (green boxes) genes.

Genes selected using a cut-off of adjusted P-Value <0.05 and |log_2_ratio| >1 applying the “Genotype contrast” are shown in **Dataset S1**. The reliability of the method was confirmed by the selection of BMP-Son of Mother Against Decapentaplegic (SMAD) target and *Tmprss6* genes. The term enrichment analysis of the genes emphasized the biological processes affected by the loss of *Tmprss6*; these comprise genes related to cytokine production, immune response to microorganisms, regulation of innate immune response and proliferation and differentiation of inflammatory cells (**Figure 2**). Enriched signaling pathways are shown in **Figure 3A** and include pathways involved in Toll like receptor signaling (KEGG pathway: mmu04620; **Figure 3B**), cytokine-cytokine receptor interaction (KEGG pathway: mmu04060; **Figure S1**) and nitric oxide metabolism (KEGG pathway: mmu00910; not shown). More in detail, loss of Tmprss6 modulates genes of the cytochrome-dependent drug metabolism (as *Cyp1a2, Cyp2b13, Cyp2b20, Cyp2b9, Cyp2c54, Cyp3a25, Cyp4a12, Cyp4b1, Cyp4f14, Cyp7a1, Cyp7b1;* KEGG pathway: mmu00980), up-regulates extracellular matrix-related genes (as *Lamb3*, *Lama1*, *Wnt*, *Ccnd1*, *Chd1*), whereas down-regulates genes involved in the response to inflammation, as *Lbp*, *Cd14*, *Tlr2*, *Myd88*, *Nfkb2* (and target genes), genes encoding for members of the chemokine ligands (as *Cxcl1*, *Cxcl2, Cxcl9, Cxcl10*) and members of the interleukine receptors (as *Il1r2*, *Il1RA*, *Il1rn*, and *Il6RA*) (**Dataset S1**).

**Figure 4:**
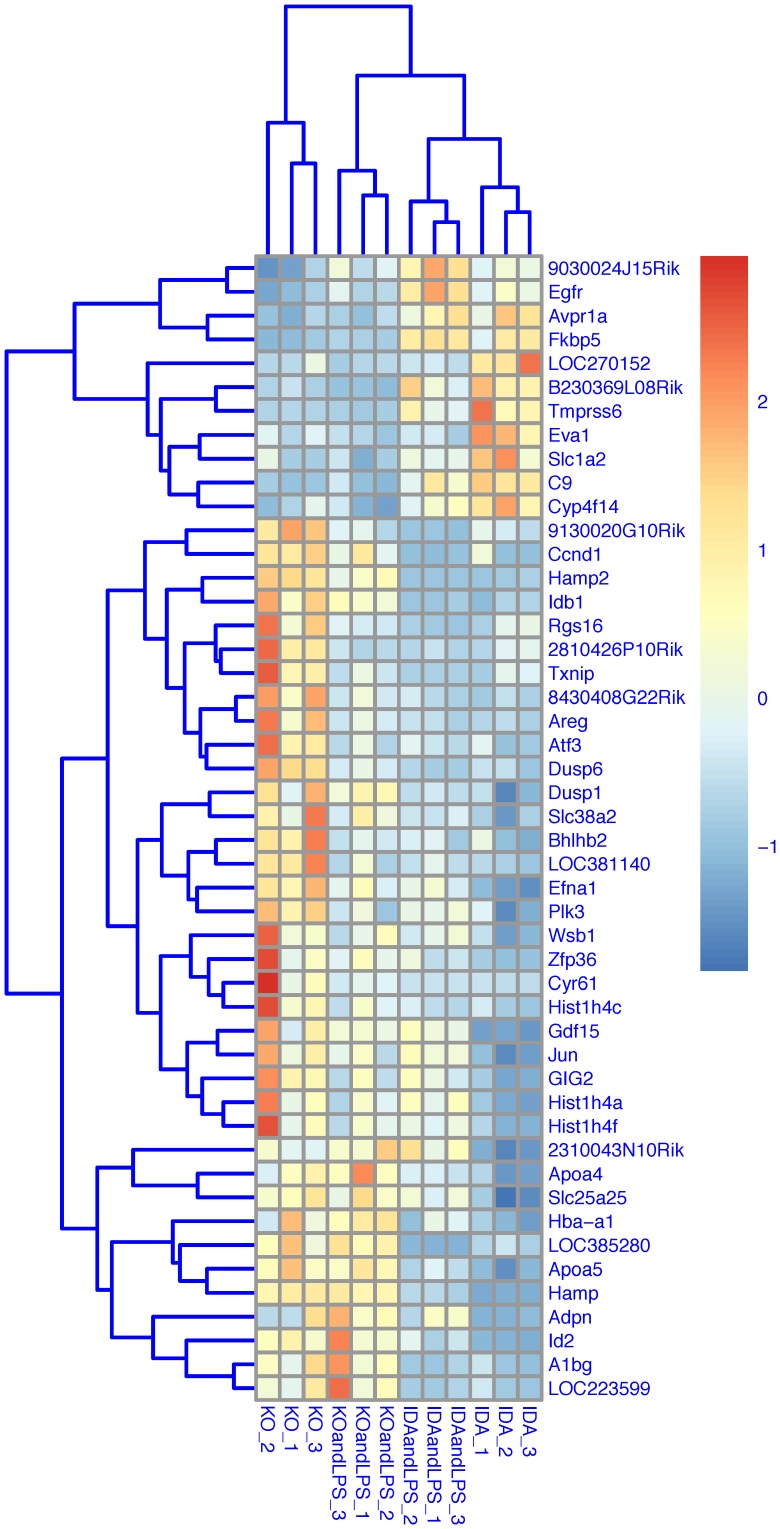
Heat map of selected genes (basal condition). The heat map represents the hierarchical clustering of 49 genes being differentially expressed according to the “Interaction contrast” (adjusted P-Value <0.05 and |log_2_ratio| >1). The expression level of each gene has been standardized by subtracting the gene’s mean expression and then dividing by the standard deviation across all samples. This scaled expression value, denoted as the Row Z-score, is plotted in red-blue scale color, with red indicating high expression.

Genes selected according to the “Genotype contrast” and significantly modulated in *Tmprss6* KO mice under basal condition are shown in **Table S1**. A selection of genes using a |log_2_ratio| >1.5 is depicted in **Table 1** **and** **Figure 4**.

**Table 1:** Genes differentially regulated under basal conditions in the liver of Tmprss6^−/−^ vs IDA mice.

Genes	Log_2_ratio^1^	Adj P-Value
Hamp	7,472	0,000007
Hamp2	4,585	0,000179
Gdf15	3,83	0,000393
Atf3	3,009	0,02693
LOC223599	2,893	0,043808
Adpn	2,581	0,023763
Efna1	2,448	0,001157
Ccnd1	2,173	0,038212
Cyr61	2,142	0,014522
8430408G22Rik	2,113	0,005684
Idb1	2,071	0,001819
Apoa4	1,914	0,020438
Slc25a25	1,872	0,013536
Id2	1,778	0,00337
Dusp6	1,768	0,000593
Jun	1,75	0,005684
Zfp36	1,723	0,018945
A1bg	1,67	0,027991
Txnip	1,65	0,029919
Hist1h4f	1,612	0,01967
Dusp1	1,61	0,029021
Hist1h4c	1,568	0,025547
9130020G10Rik	1,514	0,012299
Hist1h4a	1,51	0,015287
C9	−1,841	0,00606
Tmprss6	−1,905	0,00337

1: Log_2_ratio refers to the contrast KO.UT-IDA.UT.

**Figure 5:**
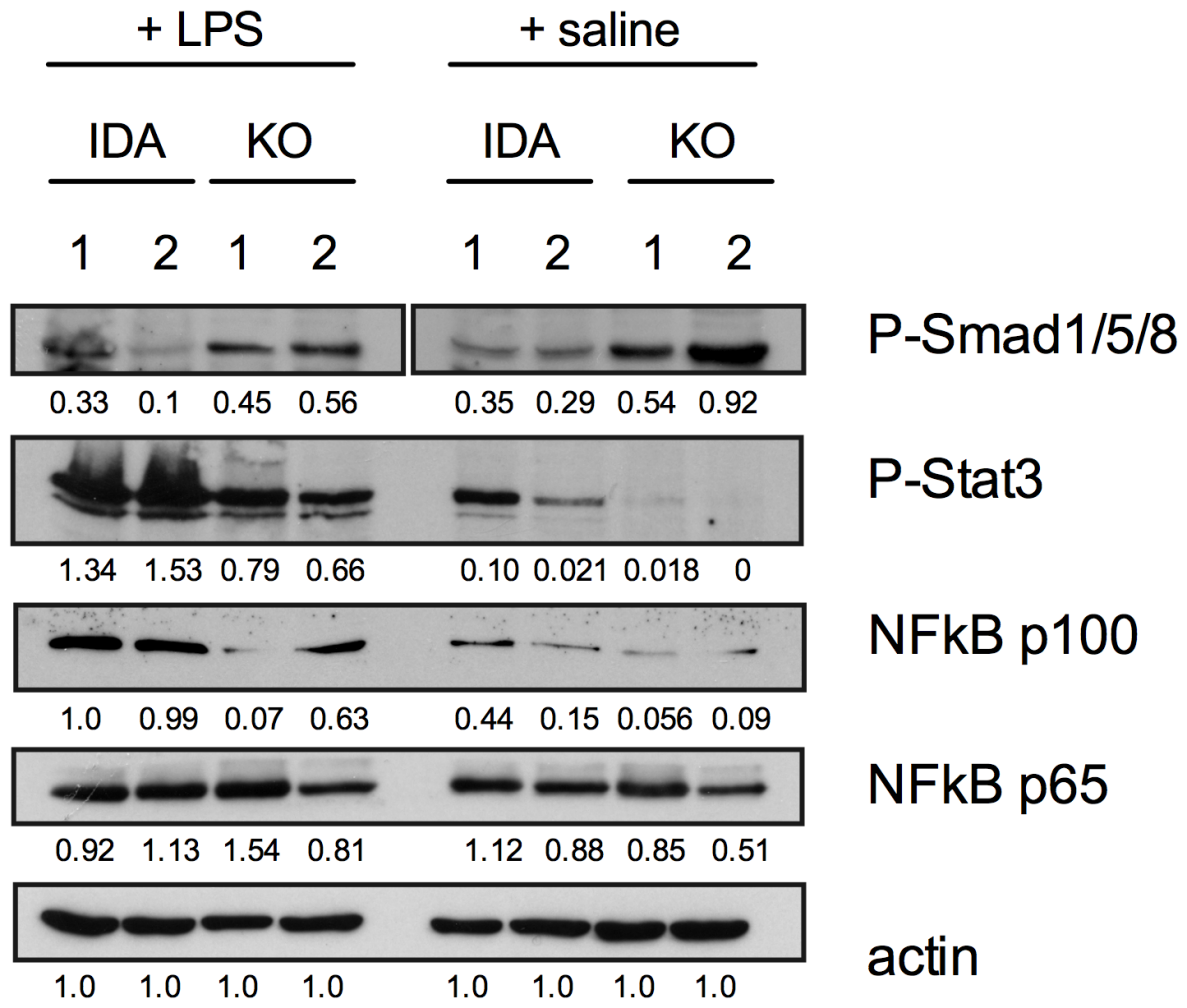
Analysis of liver BMP-SMAD, STAT3 and NF-kB proteins activation. Livers were dissociated as described in the “Material and Methods” section; extracts were subjected to SDS-PAGE and Western Blot performed using anti-Phosphorylated-SMAD1/5/8 (P-SMAD), anti-Phosphorylated-STAT3 (P-STAT3), anti-NF-kB p100, and anti-NF-kB p65. Protein levels were quantified by densitometric analysis of P-SMAD, P-STAT3, NF-kB p100 and NF-kB p65 specific bands, normalized to actin. 1 and 2 refers to liver extracts from two different mice. The numbers under the panels indicate arbitrary densitometric unit.

Up-regulation of BMP-SMAD-target genes, such as *Hamp* and *Idb1,* was previously reported in Pagani et al. [11]. Our microarray data indicate that also *Hamp2*, *Id2*, *Atoh8* and *Smad6* are up-regulated (**Dataset S1**). In agreement with the activation of the BMP-SMAD pathway, phosphorylation of SMAD1/5/8 proteins is increased in *Tmprss6* KO livers compared to IDA mice (**Figure 5**).

**Figure 6:**
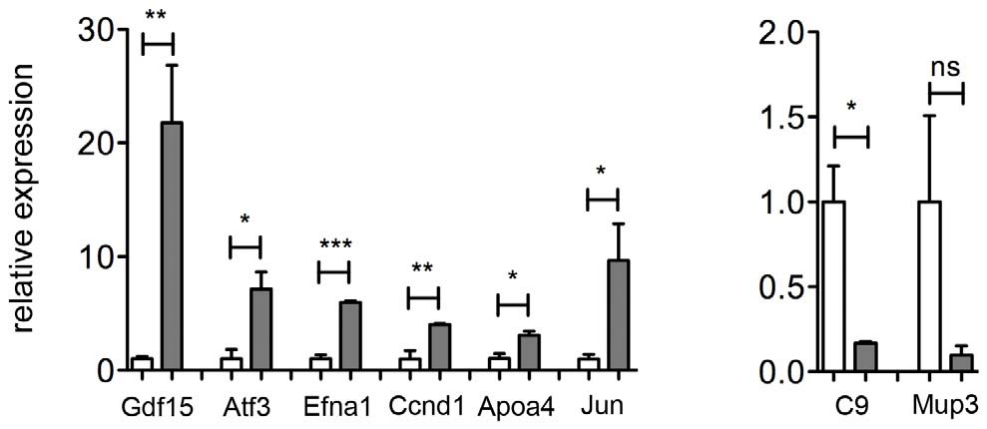
Genes differentially expressed under basal conditions. Total liver RNA was isolated from Tmprss6 KO and IDA mice. mRNA expression was quantified by TaqMan qRT-PCR. Hprt1 was used as the housekeeping gene. mRNA expression ratio was normalized to an IDA mean value of 1. Error bars indicate Standard Error.; ns, not significant; *P<0.05; **P<0.01; and ***P<0.001. White bar: IDA mice; grey bar: *Tmprss6* KO mice.

Variation of expression of representative genes (**Table 1**) was confirmed by qRT-PCR. Expression of *Gdf15*, *Atf3*, *Efna1*, *Ccdn1*, *Apoa4* and *Jun* was increased (**Figure 6** **)**, and expression of *C9*, that participates in the formation of Membrane Attack Complex and plays a key role in innate and adaptive immune response, was decreased. *Mup3* that encodes for major urinary protein 3, showed a trend towards reduction in Tmprss6 KO mice (**Figure 6**).

The expected strong impact of LPS on liver gene expression is shown in **Figure S2**. Genes modulated according to the “Treatment contrast” are related to the inflammatory response, the regulation of defense and innate immune response, cytokines production, response to DNA damage, antigen presentation and processing, T cell activation, response to viruses, bacteria and peptidoglycan, ion transport and hair cycle and follicle development.

**Figure 7:**
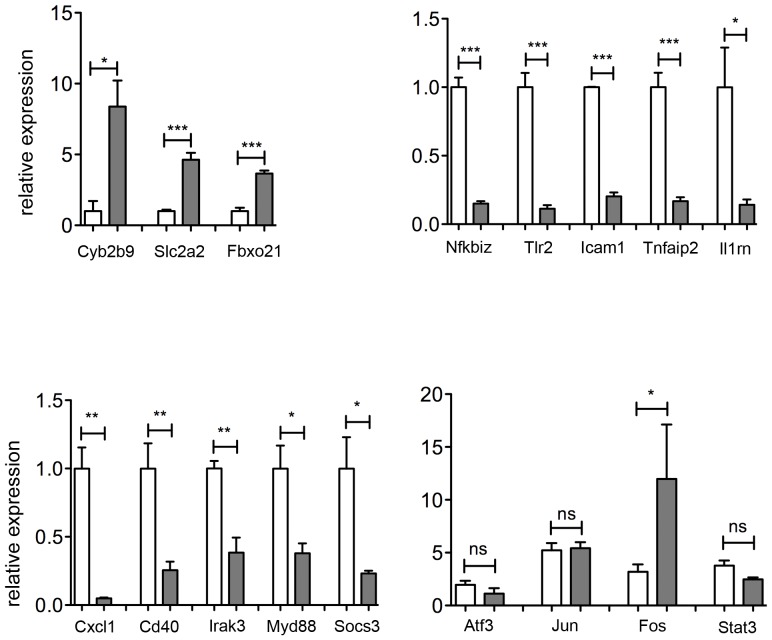
Genes differentially expressed after LPS treatment. Total liver RNA was isolated from Tmprss6 KO and IDA mice treated with LPS to induce acute inflammation (3 mice each group). TaqMan qRT-PCR was used to quantify mRNA expression and Hprt1 was used as the housekeeping gene. mRNA expression ratio was normalized to an IDA mean value of 1. Error bars indicate Standard Error.; ns, not significant; *P<0.05; **P<0.01; and ***P<0.001. White bar: IDA mice; grey bar: *Tmprss6* KO mice.

Fewer genes are modulated by LPS treatment in Tmprss6 KO mice compared to IDA animals (**Table S1**). Results of relevant genes, selected according to the “Genotype contrast” and with a |log_2_ratio| >1.5 for LPS treatment are summarized in **Table 2** **and Figure S3.** Selected genes, analyzed by qRT-PCR, are shown in **Figure 7**. Although the expression levels of these genes do not change under basal conditions (data not shown), their LPS-mediated activation is strongly impaired in *Tmprss6* KO mice. *Cyp2b9*, *Slc2a2*, *Fbxo21* and *Fos* are up-regulated in *Tmprss6* KO animals. Interestingly, the F-box protein family, that includes also Fbxo21, is involved in Iron Regulatory Protein 2 degradation by proteasome during iron-replete condition through phosphorylation-dependent ubiquitination [22], [23]. Genes involved in the regulation of the inflammatory response, as Nfkbiz, Tlr2, Icam1, Tnfaip2 and Il1rn, are strongly down-regulated in *Tmprss6* KO mice. Due to the blunted LPS response and the reduced leukocytes recruitment in *Tmprss6* KO animals [11] we analyzed the expression of liver genes involved in these signaling pathways. As shown in **Figure 7**, *Cxcl1*, *Irak3*, *Myd88* and *Socs3* are reduced in LPS-treated *Tmprss6* KO animals, confirming the impairment of the TLR-mediated signaling pathway. This evidence is further supported by the reduced expression of *Cd40*, implying the impairment of NF-kB and Stat signaling in mutant mice [24].

**Table 2:** Genes differentially regulated by LPS in the liver of Tmprss6^−/−^ vs IDA mice.

Genes	Log_2_ratio^1^	Adj P-Value	Genes	Log_2_ratio^1^	Adj P-Value
Hamp2	5,96	0,000004	Adamts4	−1,52	0,000886
Gbp1	4,613	0,000006	Ets2	−1,525	0,000056
Cyp7a1	4,387	0,000115	LOC381941	−1,527	0,000029
Cyp2b9	3,953	0,007918	Cxcl10	−1,531	0,000112
LOC223599	3,461	0,002313	Gvin1	−1,543	0,000457
EG243881	3,387	0,001134	Il1rn	−1,605	0,000258
Cyp2b13	3,184	0,025006	Zc3h12a	−1,611	0,000004
LOC385280	2,936	0,000009	Pglyrp1	−1,63	0,000009
1600032L17Rik	2,776	0,001008	Bcl3	−1,666	0,000004
Pte2a	2,598	0,000348	Upp1	−1,676	0,000025
G6pc	2,595	0,000322	Mx1	−1,69	0,000085
G0s2	2,418	0,000287	Cldn14	−1,692	0,000016
Slc40a1	2,222	0,000073	Tyki	−1,708	0,002997
Ccnd1	2,14	0,005597	Relb	−1,734	0,000006
Cyp2b20	2,102	0,002568	Samhd1	−1,741	0,000029
Gpr120	2,013	0,013532	Creld2	−1,758	0,000043
A1bg	1,934	0,001607	Gbp2	−1,784	0,000243
Hamp	1,908	0,005391	Slpi	−1,806	0,0024
D0H4S114	1,88	0,001148	Phlda1	−1,825	0,000512
BC056929	1,86	0,000187	T2bp	−1,833	0,000004
Slc2a2	1,728	0,000016	Tnfaip2	−1,885	0,000045
Cib3	1,699	0,005469	Dscr1	−1,892	0,000995
1810054O13Rik	1,659	0,000549	Icam1	−1,98	0,000004
Gal3st1	1,658	0,000159	Elf3	−2,143	0,00003
Mcc	1,633	0,000054	Cd14	−2,204	0,000566
Idb1	1,63	0,000409	IL1RA	−2,232	0,000034
Mpra	1,606	0,000081	Ifit2	−2,32	0,002581
Cbr3	1,571	0,002616	2410118P20Rik	−2,394	0,000006
Spon2	1,567	0,001891	2510004L01Rik	−2,465	0,001127
Etnk2	1,559	0,043315	Tlr2	−2,481	0,000016
Fbxo21	1,551	0,000049	Cish	−2,557	0,000031
2310047C17Rik	1,53	0,000205	Nfkbiz	−2,576	0,000004
Esm1	1,522	0,041553	Tnfaip3	−2,689	0,000004
Arrdc3	1,515	0,032914	Scara5	−2,691	0,000043
0610038K03Rik	1,508	0,000016	Cxcl2	−2,79	0,000017
4930572L20Rik	1,506	0,000102	Cxcl1	−3,111	0,000862

1: Log_2_ratio refers to the contrast KO.LPS-IDA.LPS.

Using anti-P-Stat3, we showed that Stat3 signaling was strongly decreased in *Tmprss6* KO mice both under basal condition and after LPS injection (**Figure 5**), whereas no changes were observed at the mRNA level (**Figure 7D**). Of the two NF-kB key signaling molecules (p100 and p65) only p100 is decreased in *Tmprss6* KO animals, suggesting a mild impairment of the pathway (**Figure 5**).

**Figure 8:**
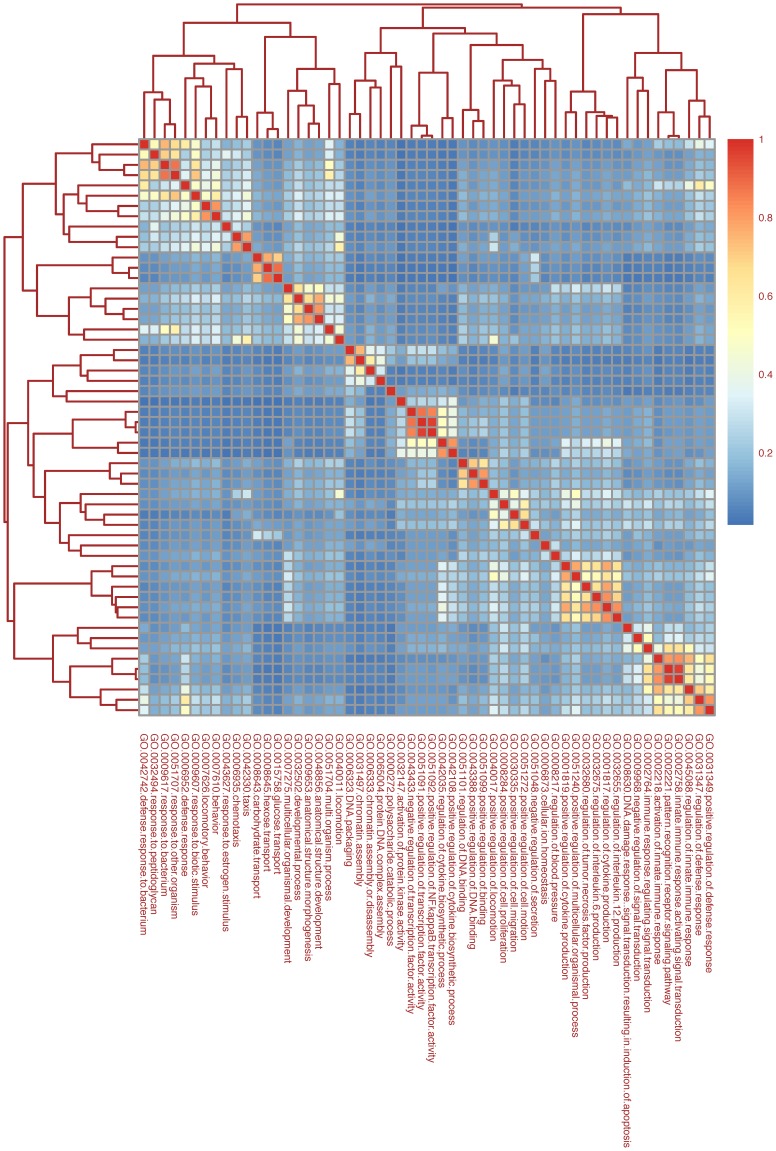
Heat map of clustered biological terms highlighted by differentially expressed genes in the “Interaction contrasts”. The heat map represents semantic similarity among gene ontology (GO) Biological Process (BP) terms. Rows and columns show the list of enriched GO BP terms derived from term enrichment analysis of Interaction significant genes. The colors represent the semantic distances calculated using GOSemSim Bioconductor package. Yellow-red clusters identify groups of terms sharing semantic similarity about biological processes.

**Figure 9:**
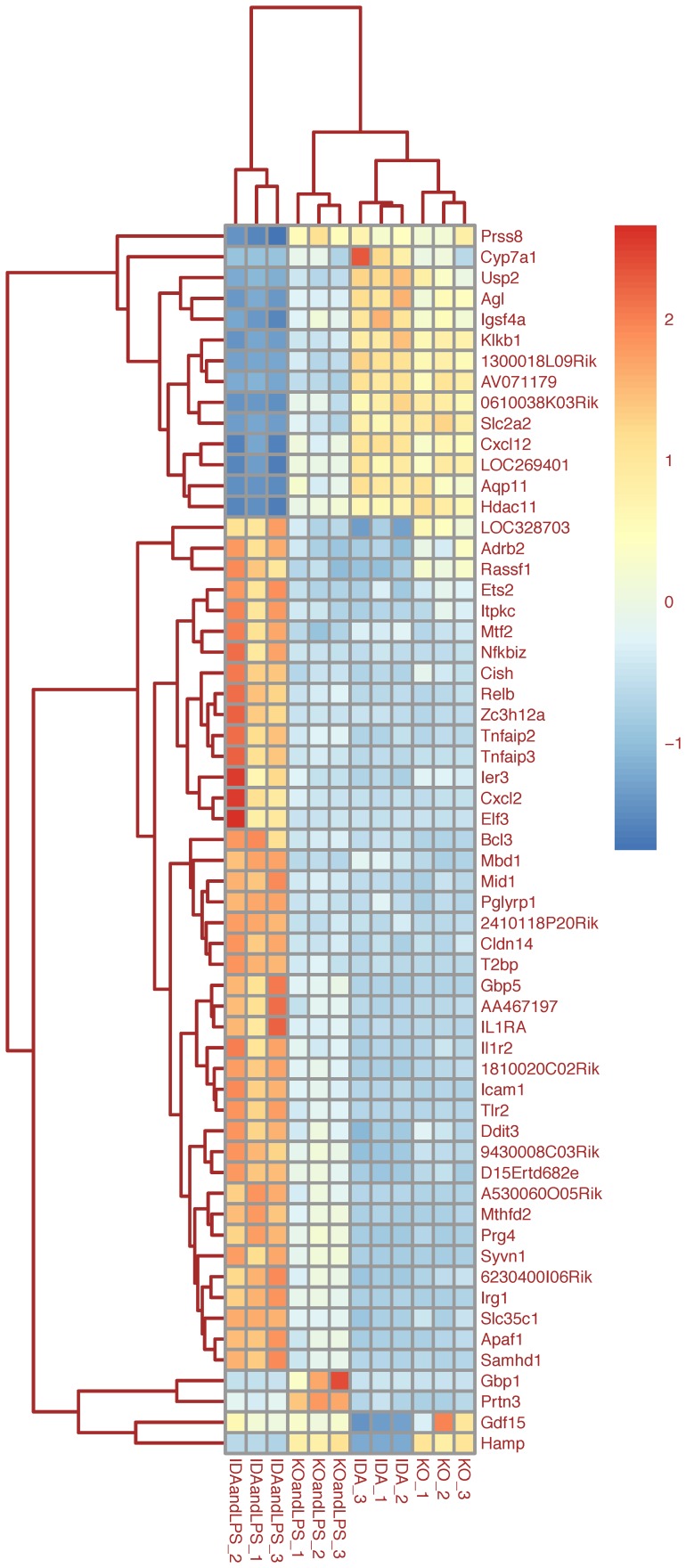
Heat map of selected genes (“Interaction contrast”). The heat map represents the hierarchical clustering of 59 genes being differentially expressed according to the “Interaction contrast” (adjusted P-Value <0.05 and |log_2_ratio| >1). The expression level of each gene has been standardized by subtracting the gene’s mean expression and then dividing by the standard deviation across all samples. This scaled expression value, denoted as the Row Z-score, is plotted in red-blue scale color, with red indicating high expression.

The “Interaction contrast” was applied to examine to what extent the genotype influences the inflammatory response and biological term enrichment analysis highlighted the LPS-related biological processes modified by the absence of Tmprss6 (**Figure 8**). These include signaling pathways related to innate immune and defense responses (*Relb* and *Nfkbiz*), production of pro-inflammatory cytokines (as *Cxcl2*, *Cxcl12*, *Il1r2*, *Il1RA*, *Tnfaip2* and *Tnfaip3*), response to bacteria and peptidoglycan (as *Tlr2*). Interestingly, genes specifically modulated in *Tmprss6* KO mice are *Hamp*, as expected, but also *Gdf15*, *Cyp7a1,* the liver specific heme enzyme that synthetizes bile acid from cholesterol, and *Prss8*, which encodes for prostasin, a GPI-anchored serine protease belonging to the type II transmembrane serine proteases as Tmprss6 (**Figure 9**).

### Expression of Immune Genes in Total Spleen *of Tmprss6* KO and IDA Mice

Together with the liver, spleen macrophages are the principal mediators of the inflammatory-anti-inflammatory response. We investigated the expression of immune-related genes in spleen of IDA and *Tmprss6* KO mice, treated or not with LPS by using the Mouse Immune Array v2.1 (AB) which allows testing >90 immune genes. Results of relevant genes selected using a cut off value of |log_2_ratio| >1.5 are shown in **Table S2.**

Compared to IDA, *Tmprss6* KO mice under basal condition up-regulate transferrin receptor 1 (*Tfrc)* and the anti-inflammatory gene *Bcl2l1*, whereas down-regulate several pro-inflammatory genes, in particular *Il1beta, Tnf alpha*, the hematopoietin cytokine family (*Il4* and *Il15*) and *Cd40lg*, which belongs to the “cytokine-cytokine receptor interaction” family. Compatible with a blunted response to LPS, nitric oxide synthase 2 (*Nos2*), pro-inflammatory cytokines (*Il6* and *Ifng*), the chemokine ligand *Cxcl11*, and molecules involved in the positive regulation of the inflammatory response (*Ptgs2* or *Cox-2*), are down-regulated in *Tmprss6* KO mice. On the other hand, few immune genes as *Bcl2l1*, *Hmox1* and *Tfrc* are up-regulated after LPS in *Tmprss6* KO mice.

To investigate whether the transcriptional changes observed in KO vs IDA spleens, after LPS challenge, were due to different basal expression levels, expression fold changes were evaluated in the two groups of mice. Most of the spleen immune genes were up-regulated at similar levels in the two groups whereas few were down-regulated in KO compared to IDA animals (**Figure S4A**). *Ccr2*, *Hmox1*, *Vegfa* and *Tfrc* showed opposite regulation (up in KO and down in IDA) (**Figure S4B**).

### Potential *Tmprss6* Target Genes

**Figure 10:**
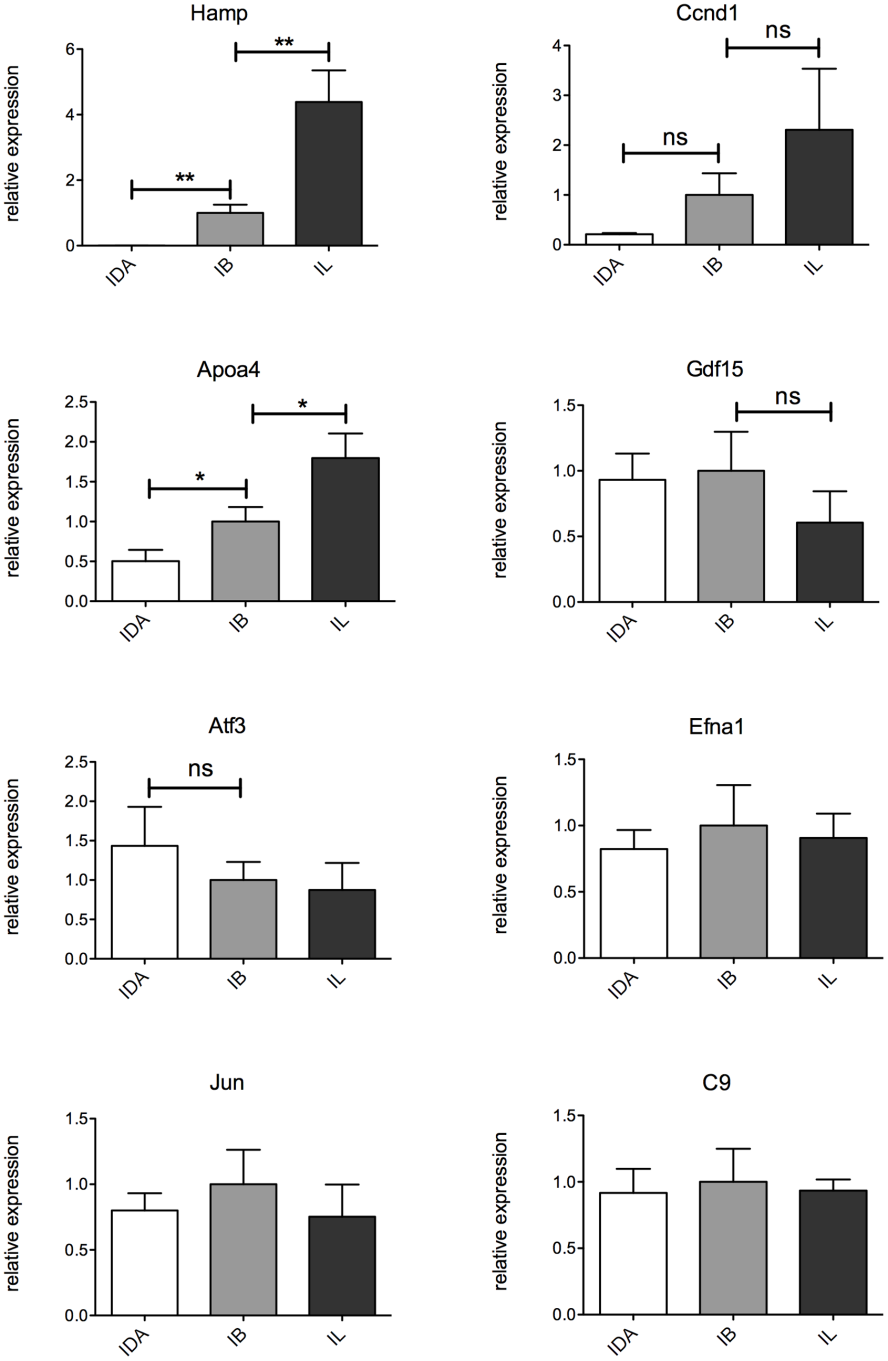
Modulation of representative genes by iron/hepcidin. 7 weeks old mice (n = 4 per group) were maintained an iron deficient (IDA, white bar), iron balanced (IB, light grey bar) and iron loaded (IL, dark grey bar) diet for 3 wks. Liver mRNA expression was measured by TaqMan qRT-PCR. Hprt1 was used as the housekeeping gene. mRNA expression ratio was normalized to an IB mean value of 1. Error bars indicate Standard Error; ns, not significant; *P<0.05 and **P<0.01.

In the attempt to distinguish whether high hepcidin or lack of *Tmprss6* determines the anti-inflammatory phenotype, we used different approaches. First we injected IDA mice with hepcidin and analyzed the expression of differentially modulated genes (Table 1). Acute hepcidin treatment strongly decreases *Gdf15, Atf3, and Efna1* expression 8 hours post-injection. *C9* is only slightly down-regulated, whereas *Apoa4* is increased by hepcidin injection (**Figure S5**). To investigate the modulation of the same genes in chronic conditions of low and high hepcidin, we studied their liver expression in iron deficient (IDA), iron balanced (IB) and iron loaded (IL) wild type mice. As expected, hepcidin was down- and up-regulated in iron deficiency and overload respectively, and genes such as *Ccdn1* and *Apoa4* were modulated according to the iron/hepcidin levels (**Figure 10**), as already reported [25]. However, *Gdf15, Atf3, Efna1*, *Jun* and *C9* expression remained unchanged even in IL mice, excluding that high hepcidin is responsible for variation of these genes in *Tmprss6* KO mice (**Figure 10**).

## Discussion

Hepcidin is a liver “defensin-like” acute phase protein with anti-microbial activity *in vitro* and potentially *in vivo* due to its ability to decrease plasma iron, a growth factor for invading pathogens. In response to inflammation, not only liver but also macrophages strongly increase hepcidin production, amplifying iron retention through the autocrine effect of hepcidin on macrophage ferroportin [26]. The inflammation-mediated hepcidin regulation has clinical relevance, since macrophage iron sequestration results in iron restricted erythropoiesis and Anemia of Chronic Disease (ACD), a common type of anemia observed in infections and acute and chronic inflammatory disorders as an adaptation mechanism. The anti-inflammatory role of hepcidin might not only be limited to intracellular iron sequestration. Changes in hepcidin (and/or of iron levels) might modulate the inflammatory response in vivo. Although the kinetics and doses of LPS were different in the different experimental settings, mouse models characterized by low hepcidin and iron overload, as *Hfe* ^−/−^ [27] and *hepcidin* ^−/−^ mice [6], up-regulate inflammatory genes in response to LPS more actively than wild type animals. Moreover, IL6 treatment of liver conditional *Smad4* deficient mice [28] strongly induced liver acute phase proteins expression (*Crp* and *Saa-1*), compared to control animals. On the contrary, *Tmprss6* KO mice, characterized by iron deficient anemia and high hepcidin, have a blunted inflammatory response compared to mice with a diet-induced iron deficient anemia and low hepcidin [11].

The genome-wide expression profiling of *Tmprss6* KO mice livers, compared to IDA animals, revealed the downregulation of genes involved in immune response, suggesting that high hepcidin and/or absence of *Tmprss6* are associated with an anti-inflammatory phenotype. The evidence of a decreased STAT3 and NF-kB signaling, both at RNA and protein levels, further supports this finding, strengthening the pro-inflammatory role of Tmprss6, compatible with the finding of a pro-inflammatory condition in iron deficiency anemia [11], a condition characterized by TMPRSS6 activation [29] [30]. As additional validation of this profiling, a further analysis has been carried out using the Gene Set Enrichment Analysis (GSEA) [20]. Gene sets related to inflammation and immune response are significantly enriched among the negative correlated genes (GSEA Analysis S1).

Liver gene expression profile of *Tmprss6* KO mouse is available in the literature [9], however the experiment was performed on a single animal using a wild type iron replete animal as control, and thus is not comparable to our analysis.

Due to the genetic loss of the BMP pathway physiological inhibitor [31], some BMP-SMAD target genes are up-regulated in *Tmprss6* KO mice. Indeed in these mice we show an increased phosphorylation of SMAD1/5/8, expected to be low in conditions of iron deficiency. Other up-regulated genes, such as *Gdf15*, *Atf3* and *Jun*, are linked to an anti-inflammatory response. *Gdf15*, a secreted member of the transforming growth factor (TGF)-beta superfamily highly expressed in liver tissue and in erythroid precursors, is commonly up-regulated during inflammation and exerts its function through phosphorylation of SMAD2 and SMAD3 [32]. *Gdf15* inhibits leukocyte integrin activation thus reducing inflammatory cell recruitment after myocardial infarction [33], a finding in agreement with the reduced leukocytes recruitment observed in LPS-treated *Tmprss6* KO mice [11]. Although *Gdf15* is activated by inflammation, *Tmprss6* KO mice do not show increased inflammatory cytokines, suggesting that in these mice *Gdf15* is up-regulated by an inflammation-independent mechanism. *Gdf15* has been proposed also as a hepcidin inhibitor [34]. However, the high hepcidin levels suggest that either *Tmprss6* KO mice lack *Gdf15* target molecule(s) or that *Gdf15* does not inhibit hepcidin *in vivo*, as recently proposed [35]. *Atf3* is a member of the Activation Transcription Factor family, which represses IL-6, IL-12 and other cytokines downstream Toll-like receptor 4 (TLR4) and provides a negative feedback to prevent excessive inflammation [36]. *Atf3* is activated by inflammation and by the TGF-beta mediator Smad3 [37], suggesting a functional connection with *Gdf15*.

The transcription factor *Jun* participates with other proteins (Fos, ATF and JDP) in the formation of activator protein 1 (AP-1) complex, essential in regulating gene expression in response to a variety of stimuli as cytokines, growth factors, stress, and infections. Functional cross-talk between TGF-beta, SMAD proteins and Jun has been demonstrated: the SMAD3/SMAD4 heterodimer acts synergistically with the Jun/Fos heterodimer to activate transcription in response to TGF-beta [38]. This finding is of interest since *Gdf15, Atf3* and *Jun*, all participate to the AP-1 complex formation.

In the attempt to distinguish whether the observed transcriptional changes are due to the genetic loss of *Tmprss6* and/or to the differential regulation of the *BMP-SMAD*-target genes, as hepcidin, we analyzed the expression of some representative genes (**Figure 10** and **Figure S5**) in conditions of acute and chronic high hepcidin. Acute hepcidin injection in IDA mice does not substantially change the expression of the studied genes, and even strongly down-regulates *Gdf15* and *Jun.*, Dietary iron loaded mice with chronic high hepcidin do not modulate the expression of the representative genes, excluding a role for hepcidin in their regulation.

Our approach of comparing *Tmprss6* KO mice with IDA animals eliminates the contribution of iron deficiency to the modulation of gene expression. This allows an interesting comparison with published data on the opposite model of *Hfe* hemochromatosis [25]. The liver expression profiling of the *Hfe^−/−^* mice was analyzed versus iron-loaded animals, excluding transcriptional changes due to iron overload. Comparing *Tmprss6* KO and *Hfe^−/−^* liver transcriptomes reveals interesting opposite expression of specific genes, as shown in **Table S3**. First, some inflammation related genes, such as Il6ra and acute phase proteins (*Mup4, Saa1, Saa2, Saa3),* down-regulated or unchanged in *Tmprss6* KO mice are up-regulated in *Hfe^−/−^* mice. On the contrary, *Hamp1*, genes participating to the stress response (*Egr1* and Gadd45g) and immune genes (suppressors of cytokine signaling and histocompatibility class II Ag) are down-regulated in *Hfe^−/−^* but not in *Tmprss6* KO animals. Considering that the BMP-SMAD pathway is attenuated in *Hfe−/−* [39] and strongly activated in *Tmprss6* KO mice it is tempting to speculate that these differences are related to the activity of this pathway. Further studies are needed to verify this hypothesis.

## Supporting Information

Figure S1 **Representations of KEGG pathways enriched in the “genotype contrast”.** Representation of the KEGG signaling pathway Cytokine-Cytokine Receptor Interaction, showing up-regulated (red boxes) and down-regulated (green boxes) genes
(TIFF)

Figure S2 **Heat map of clustered biological terms highlighted by differentially expressed genes in the “Treatment contrast”.** The heat map represents semantic similarity among gene ontology (GO) Biological Process (BP) terms. Rows and columns show the list of enriched GO BP terms derived from term enrichment analysis of Treatment significant genes. The colors represent the semantic distances calculated using GOSemSim Bioconductor package. Yellow-red clusters identify groups of terms sharing semantic similarity about biological processes.
(TIFF)

Figure S3 **Heat map of genes modulated by LPS treatment.** The heat map represents the hierarchical clustering of 72 genes being differentially expressed according to the “genotype contrast” and the pair-wise comparison KO.LPS-IDA.LPS (adjusted P-Value <0.05 and |log_2_ratio| >1.5). The expression level of each gene has been standardized by subtracting that gene’s mean expression and then dividing by the standard deviation across all samples. This scaled expression value, denoted as the Row Z-score, is plotted in red-blue scale color, with red indicating high expression.
(TIFF)

Figure S4 **Overlap in immune spleen genes after LPS treatment. A**) Venn diagrams represent overlap in immune genes significantly up-regulated (left panel) or down-regulated (right panel) in the spleen of Tmprss6 KO and IDA mice upon LPS challenge. **B**) List of immune genes selectively up-regulated (left panel) or down-regulated (right panel) only in one group of mice.
(TIF)

Figure S5 **Transcriptional modulation of representative liver genes by acute hepcidin treatment.** TaqMan qRT-PCR was used to analyze gene expression in the liver of 7 wks old mice IDA mice pretreated with hepcidin (100 µg) or vehicle (n = 4 per group). Hprt1 was used as housekeeping gene to normalize gene expression. mRNA expression ratio was normalized to an IDA (-hepcidin) mean value of 1. ns: not significant; **: P<0.01; ***; P<0.001. White bar: vehicle-injected IDA mice; grey bar: hepcidin-injected IDA mice.
(TIF)

GSEA Analysis S1 **Gene Set Enrichment Analysis (GSEA) analysis of “Genotype” significant genes.**
(DOCX)

Table S1 **Summary of the differential expression results.**
(DOCX)

Table S2 **Analysis of selected immune genes in spleen of** ***Tmprss6*** **^−/−^ compared to IDA mice.**
(DOCX)

Table S3 **Comparison between** ***Tmprss6*** **KO and** ***Hfe*** **−/− on the expression of selected liver genes.**
(DOCX)

Table S4 **List of oligonucleotides primers used for qRT-PCR.**
(DOCX)

Dataset S1 **Limma modeling expression data.** The table listed the results obtained by limma modeling and testing for the differential expression using the following contrasts: KO.UT-ID.UT; KO.LPS-ID.LPS; ID.LPS-ID.UT; KO.LPS-KO.UT.
(XLSX)
