# Supplementary figures and images for: A Strong Anti-Inflammatory Signature Revealed by Liver Transcription Profiling of Tmprss6−/− Mice
Source: PLoS One. 2013 Jul 29;8(7):e69694. doi: 10.1371/journal.pone.0069694 (PMC3726786; doi:10.1371/journal.pone.0069694)

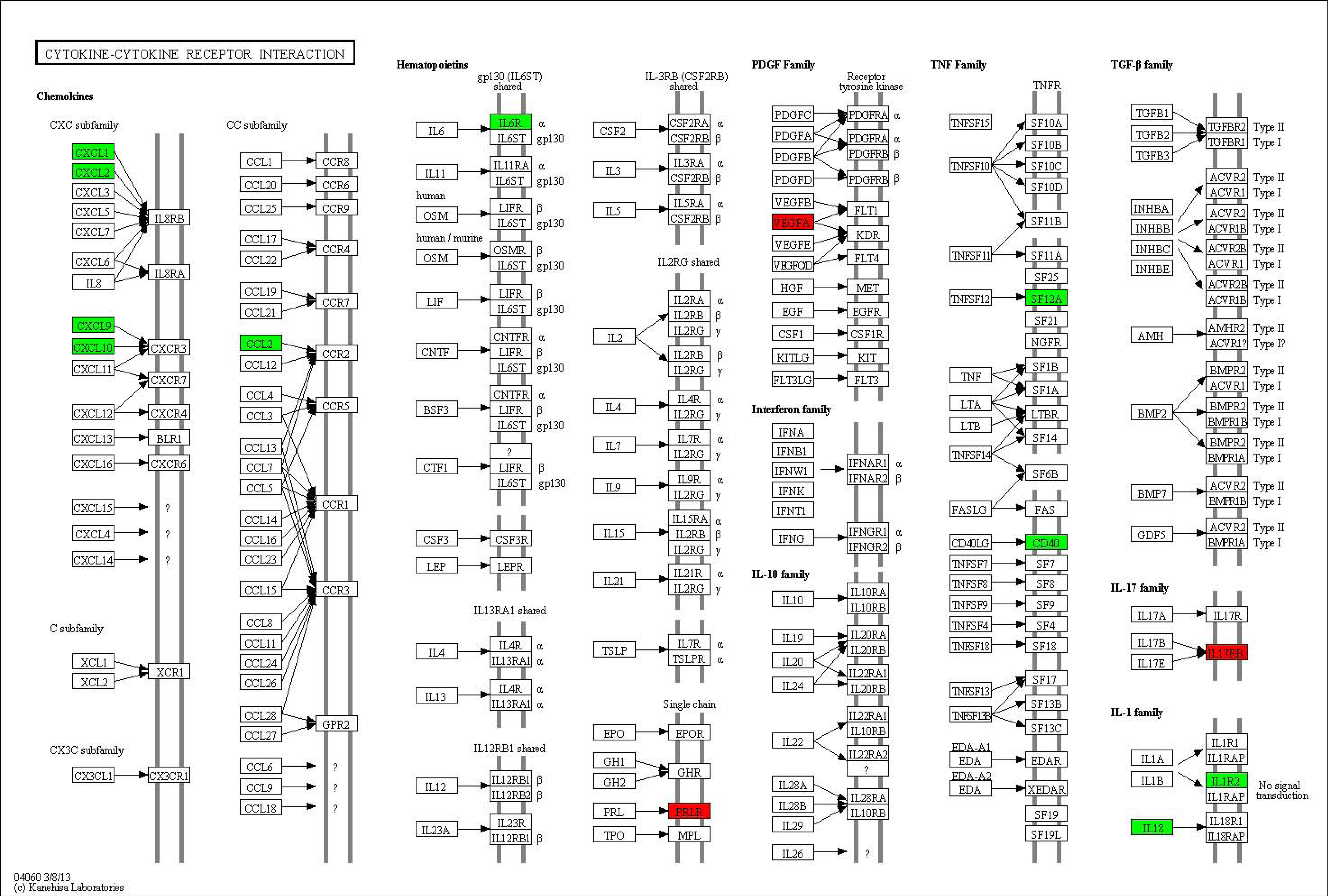

Supplement: Figure S1 — Representations of KEGG pathways enriched in the “genotype contrast”. Representation of the KEGG signaling pathway Cytokine-Cytokine Receptor Interaction, showing up-regulated (red boxes) and down-regulated (green boxes) genes (TIFF) [file pone.0069694.s001.tiff]

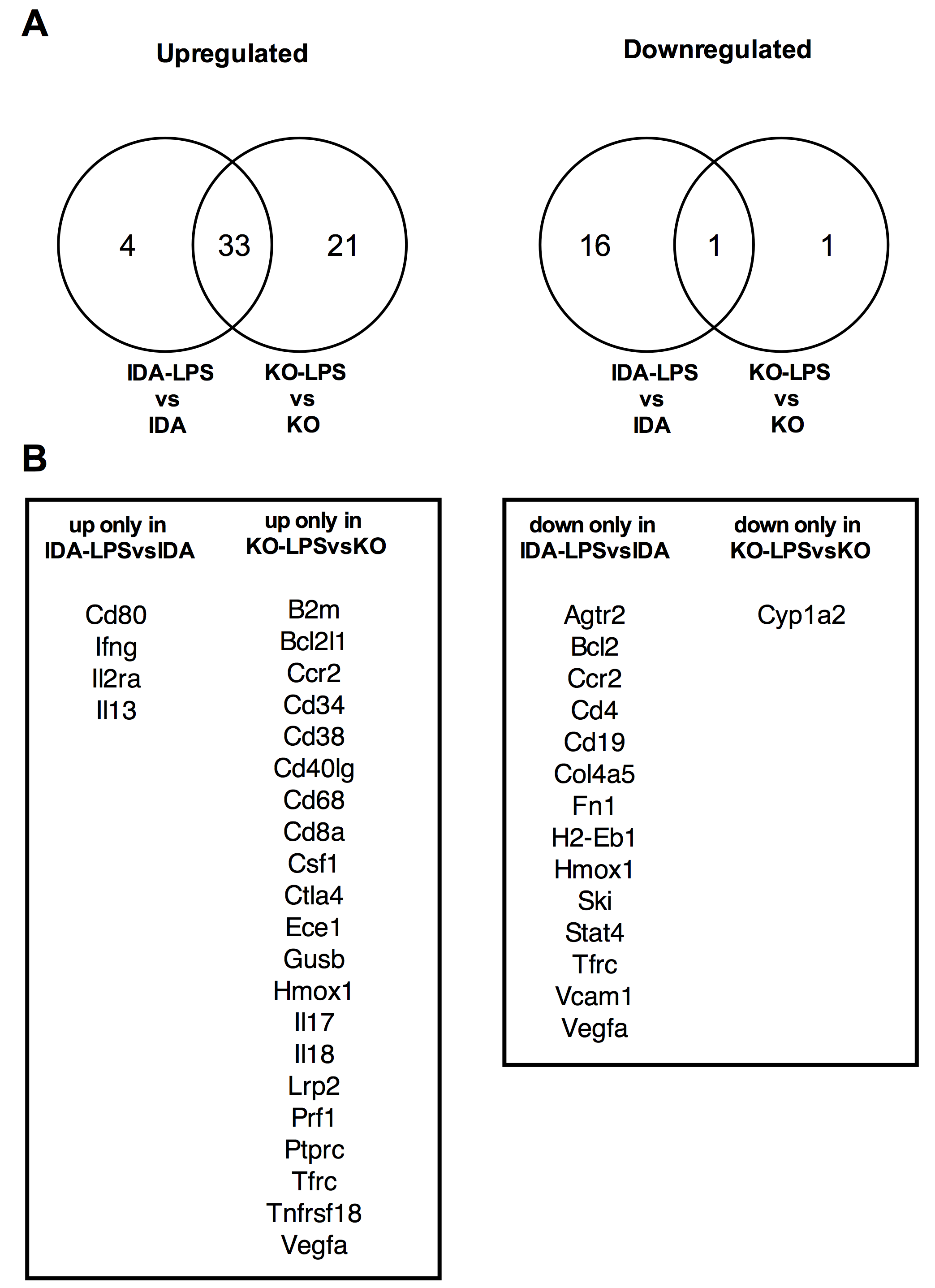

Supplement: Figure S4 — Overlap in immune spleen genes after LPS treatment. A) Venn diagrams represent overlap in immune genes significantly up-regulated (left panel) or down-regulated (right panel) in the spleen of Tmprss6 KO and IDA mice upon LPS challenge. B) List of immune genes selectively up-regulated (left panel) or down-regulated (right panel) only in one group of mice. (TIF) [file pone.0069694.s004.tif]

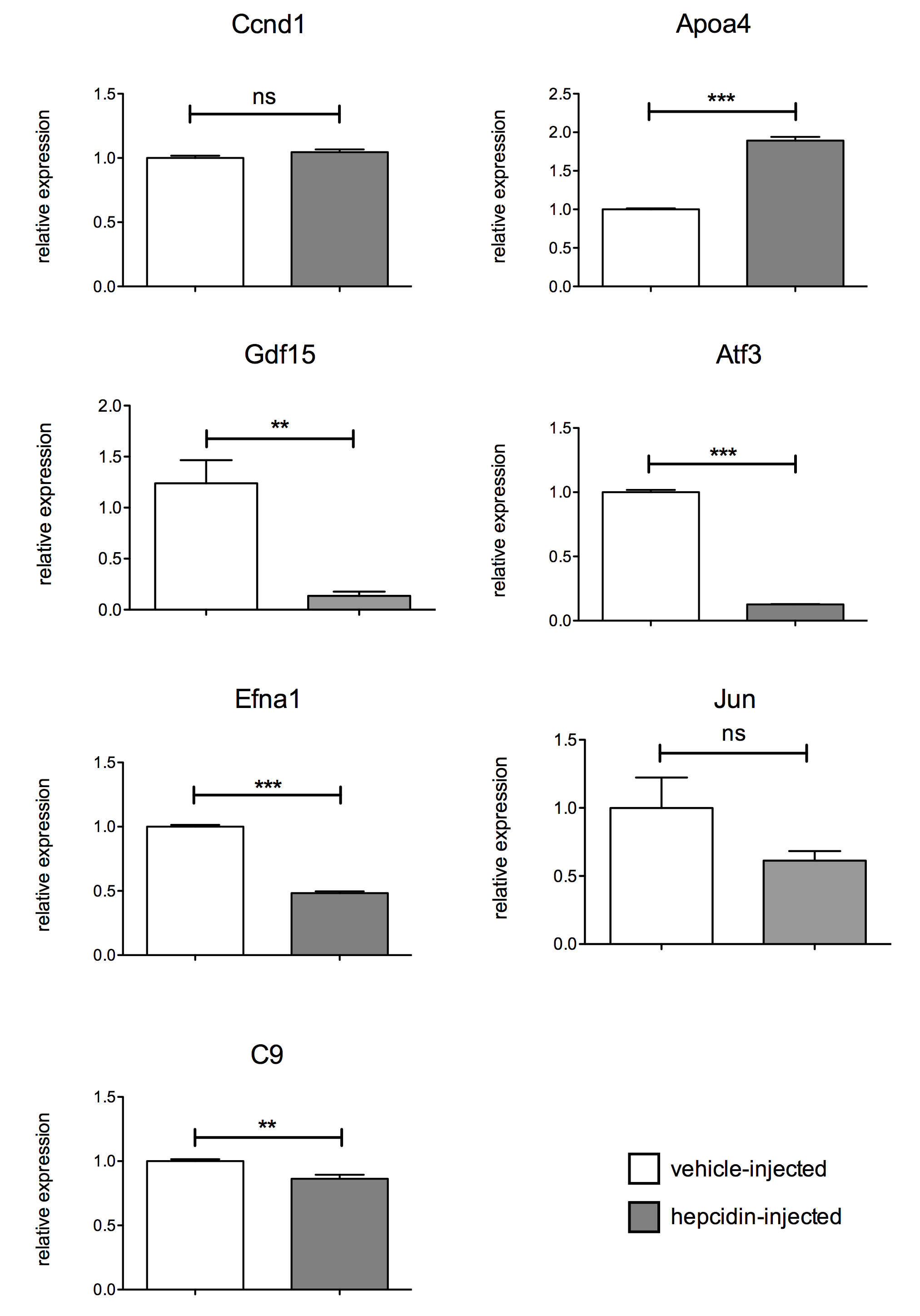

Supplement: Figure S5 — Transcriptional modulation of representative liver genes by acute hepcidin treatment. TaqMan qRT-PCR was used to analyze gene expression in the liver of 7 wks old mice IDA mice pretreated with hepcidin (100 µg) or vehicle (n = 4 per group). Hprt1 was used as housekeeping gene to normalize gene expression. mRNA expression ratio was normalized to an IDA (-hepcidin) mean value of 1. ns: not significant; **: P<0.01; ***; P<0.001. White bar: vehicle-injected IDA mice; grey bar: hepcidin-injected IDA mice. (TIF) [file pone.0069694.s005.tif]
