# Supplementary material for: A Strong Anti-Inflammatory Signature Revealed by Liver Transcription Profiling of Tmprss6−/− Mice
Source: PLoS One. 2013 Jul 29;8(7):e69694. doi: 10.1371/journal.pone.0069694 (PMC3726786; doi:10.1371/journal.pone.0069694)
Supplement: GSEA Analysis S1 — Gene Set Enrichment Analysis (GSEA) analysis of “Genotype” significant genes. (DOCX) [file pone.0069694.s006.docx]

***GSEA analysis of “Genotype” significant genes***

Gene Set Enrichment Analysis (GSEA) [[1](#_ENREF_1)], which identifies groups of genes enriched towards the top or bottom of a ranked list of genes based on a running sum statistic, has been used to identify functionally related groups of genes whose expression pattern was correlated with the template defined by the C2 curated gene set from MSigDB [[1](#_ENREF_1)], regarding chemical and genetic perturbations [[1](#_ENREF_1)]. “Genotype” significant terms have been used to perform the enrichment analysis. This analysis gave qualitatively very similar results to those obtained using DAVID[[2](#_ENREF_2)].

At the default FDR P-Value cut-off within GSEA of 0.25, 41 gene sets showed significant enrichment in the following **Table**.

| **NAME** | **FDR q-val** |
| --- | --- |
| ALTEMEIER_RESPONSE_TO_LPS_WITH_MECHANICAL_VENTILATION | 0 |
| SEKI_INFLAMMATORY_RESPONSE_LPS_UP | 0 |
| PEDRIOLI_MIR31_TARGETS_DN | 0 |
| ZHANG_RESPONSE_TO_IKK_INHIBITOR_AND_TNF_UP | 4,49E-04 |
| ZHOU_INFLAMMATORY_RESPONSE_LPS_UP | 5,62E-04 |
| ICHIBA_GRAFT_VERSUS_HOST_DISEASE_D7_UP | 5,64E-04 |
| GRAESSMANN_APOPTOSIS_BY_SERUM_DEPRIVATION_UP | 0,002316428 |
| RASHI_RESPONSE_TO_IONIZING_RADIATION_2 | 0,005067661 |
| GRAESSMANN_RESPONSE_TO_MC_AND_SERUM_DEPRIVATION_UP | 0,00534636 |
| ZHOU_INFLAMMATORY_RESPONSE_FIMA_UP | 0,015026657 |
| CHARAFE_BREAST_CANCER_LUMINAL_VS_BASAL_DN | 0,0168102 |
| GALINDO_IMMUNE_RESPONSE_TO_ENTEROTOXIN | 0,02875455 |
| REACTOME_CYTOKINE_SIGNALING_IN_IMMUNE_SYSTEM | 0,03045428 |
| ZWANG_CLASS_3_TRANSIENTLY_INDUCED_BY_EGF | 0,03284944 |
| ACEVEDO_FGFR1_TARGETS_IN_PROSTATE_CANCER_MODEL_UP | 0,03377026 |
| MCLACHLAN_DENTAL_CARIES_UP | 0,039672695 |
| ZHOU_INFLAMMATORY_RESPONSE_LIVE_UP | 0,041062746 |
| MCLACHLAN_DENTAL_CARIES_DN | 0,042074595 |
| ACEVEDO_LIVER_TUMOR_VS_NORMAL_ADJACENT_TISSUE_UP | 0,04970385 |
| ONDER_CDH1_TARGETS_2_DN | 0,050082497 |
| HORIUCHI_WTAP_TARGETS_UP | 0,05550856 |
| MIKKELSEN_ES_ICP_WITH_H3K4ME3 | 0,069666125 |
| MARKEY_RB1_ACUTE_LOF_UP | 0,07076531 |
| OSWALD_HEMATOPOIETIC_STEM_CELL_IN_COLLAGEN_GEL_UP | 0,07730035 |
| FOSTER_TOLERANT_MACROPHAGE_DN | 0,091524735 |
| MARTENS_BOUND_BY_PML_RARA_FUSION | 0,10039572 |
| BERTUCCI_MEDULLARY_VS_DUCTAL_BREAST_CANCER_UP | 0,11027289 |
| HAN_SATB1_TARGETS_DN | 0,11276612 |
| REACTOME_IMMUNE_SYSTEM | 0,11519858 |
| YANG_BCL3_TARGETS_UP | 0,116795816 |
| BOQUEST_STEM_CELL_CULTURED_VS_FRESH_UP | 0,118457295 |
| SENESE_HDAC1_TARGETS_UP | 0,17709301 |
| RUTELLA_RESPONSE_TO_CSF2RB_AND_IL4_DN | 0,17863813 |
| PHONG_TNF_RESPONSE_NOT_VIA_P38 | 0,1842554 |
| HIRSCH_CELLULAR_TRANSFORMATION_SIGNATURE_UP | 0,19029449 |
| GOZGIT_ESR1_TARGETS_DN | 0,1935991 |
| RUTELLA_RESPONSE_TO_HGF_VS_CSF2RB_AND_IL4_UP | 0,19487873 |
| QI_PLASMACYTOMA_UP | 0,19691738 |
| ENK_UV_RESPONSE_EPIDERMIS_UP | 0,24534228 |
| NUYTTEN_EZH2_TARGETS_UP | 0,24687678 |
| CHEN_METABOLIC_SYNDROM_NETWORK | 0,24838467 |

Gene sets related to inflammation and immune response proved to be significantly enriched among the negative correlated genes, as shown by the enrichment plots in **Figures 1A and 1B**

A leading-edge subset analysis has been performed as well, aiming at finding genes, which drive the enrichment results, considering the 41 significant gene sets (**Figure 2**)

**Experimental Procedures**

A pre-ranked analysis has been performed using 508 “Genotype” significant genes derived from LIMMA analysis towards c2-2.all.v3.1.symbols belonging to the C2 curated gene set database MSigDB, regarding chemical and genetic perturbations. This database contains 4850 gene sets. The pre-ranked analysis has been performed using default parameters (Categories with fewer than 15, or greater than 500 members, were excluded from the analysis, 1000 permutations have been done). Leading edge analysis has been performed on the 41 significant gene sets with default parameters.

**References**

1. Subramanian A, Tamayo P, Mootha VK, Mukherjee S, Ebert BL, et al. (2005) Gene set enrichment analysis: a knowledge-based approach for interpreting genome-wide expression profiles. Proceedings of the National Academy of Sciences of the United States of America 102: 15545-15550.

2. Huang da W, Sherman BT, Lempicki RA (2009) Systematic and integrative analysis of large gene lists using DAVID bioinformatics resources. Nat Protoc 4: 44-57.

**Legend to Figures**

**Figure 1.** Profile of the Running Enrichment Score & Positions of Gene Set Members on the Rank Ordered List. A computation of overlaps of this enriched set towards C5 database in MSigDB shows a significant representation of gene sets of Inflammatory response (data not shown). **A) The** enrichment plot “ALTEMEIER RESPONSE TO LPS WITH MECHANICAL VENTILATION” regards genes up-regulated in lung tissue upon LPS aspiration with mechanical ventilation (MV) compared to control (PBS aspiration without MV). **B)** The enrichment plot “SEKI INFLAMMATORY RESPONSE LPS UP” represents genes up-regulated in hepatic stellar cells after stimulation with bacterial lipopolysacharide (LPS).

**Figure 2.**. Heat map of clustered genes in the leading edge subsets. In the heat map the expression log_2_ratio are represented as colors, where the range of color (red-blue) shows the range of expression values (high-low). Genes and gene sets are represented in rows and columns, respectively.

**Figure 1**

**A**


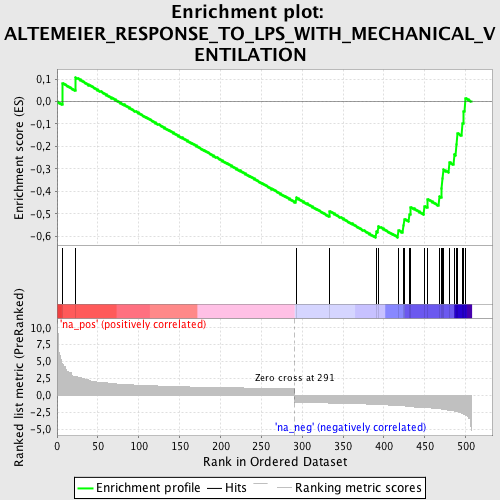


**B**


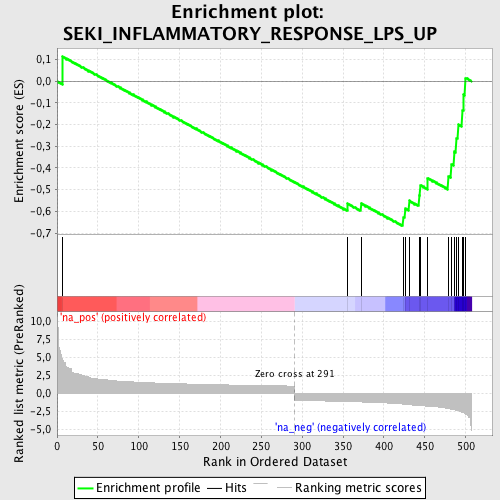


**
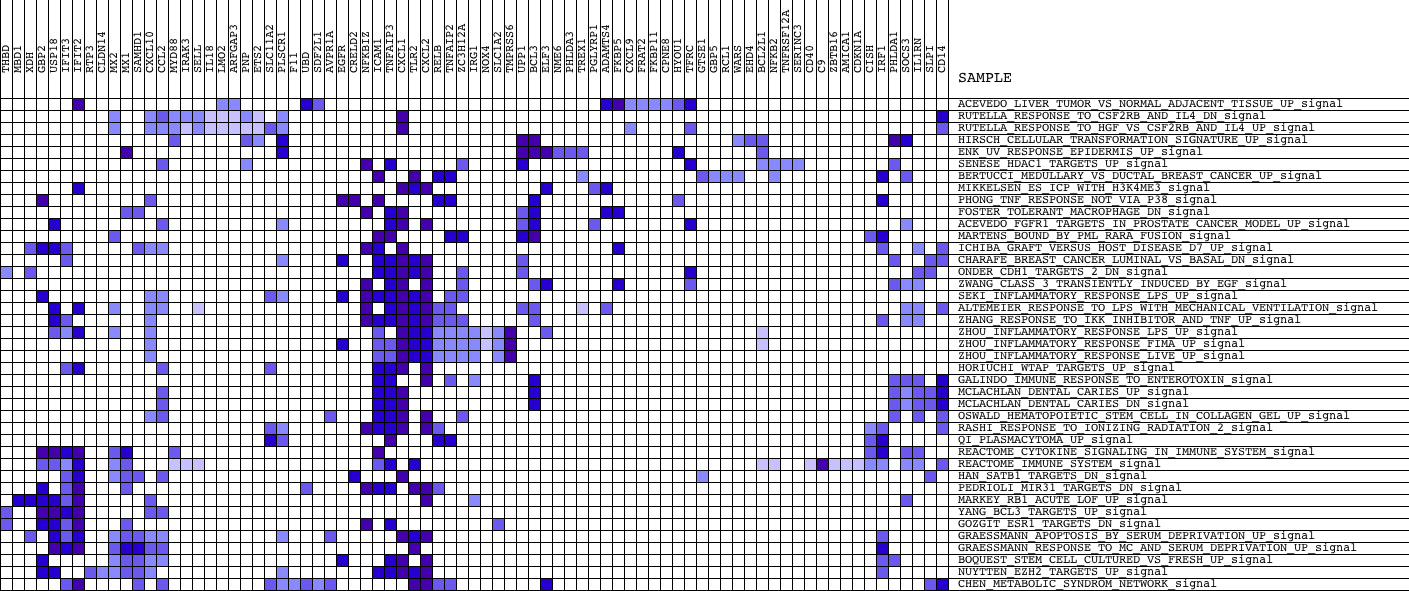
Figure 2**
