# Supplementary material for: A Strong Anti-Inflammatory Signature Revealed by Liver Transcription Profiling of Tmprss6−/− Mice
Source: PLoS One. 2013 Jul 29;8(7):e69694. doi: 10.1371/journal.pone.0069694 (PMC3726786; doi:10.1371/journal.pone.0069694)
Supplement: Table S1 — Summary of the differential expression results. (DOCX) [file pone.0069694.s007.docx]

Table S1. Summary of the differential expression results.

|  | Genotype | Treatment | Interaction | KO  vs IDA | KO  vs IDA (LPS) | LPS-IDA vs  IDA | LPS-KO vs KO |
| --- | --- | --- | --- | --- | --- | --- | --- |
| Up-regulated | 291 | 939 | 180 | 52 | 134 | 381 | 61 |
| Down-regulated | 217 | 910 | 85 | 11 | 159 | 521 | 206 |
| Unchanged | 10146 | 8805 | 10389 | 10591 | 10361 | 9752 | 10387 |
