# Supplementary material for: A Strong Anti-Inflammatory Signature Revealed by Liver Transcription Profiling of Tmprss6−/− Mice
Source: PLoS One. 2013 Jul 29;8(7):e69694. doi: 10.1371/journal.pone.0069694 (PMC3726786; doi:10.1371/journal.pone.0069694)
Supplement: Table S2 — Analysis of selected immune genes in spleen of Tmprss6 −/− compared to IDA mice. (DOCX) [file pone.0069694.s008.docx]

Table S2. Analysis of selected immune genes in spleen of *Tmprss6*^-/-^ vs IDA mice

| **Treatment** | **Genes** | **Description** | **Log_2_ratio** |
| --- | --- | --- | --- |
| saline | Tfrc | transferrin receptor 1 | 2.2 |
|  | Bcl2l1 | bcl2-like 1 | 1.5 |
|  | Il1b | interleukin 1 beta | -1.5 |
|  | Tnf | tumor necrosis factor alpha | -1.6 |
|  | Il15 | interleukin 15 | -1.7 |
|  | Il4 | interleukin 4 | -1.7 |
|  | Cd40lg | cd40 ligand | -2.0 |
|  | Lrp2 | low density lipoprotein receptor-related protein 2 | -3.1 |
| LPS | Tfrc | transferrin receptor 1 | 3,5 |
|  | Bcl2l1 | bcl2-like 1 | 1,7 |
|  | Hmox1 | heme oxygenase 1 | 1,5 |
|  | Il13 | interleukin 13 | -1,6 |
|  | Cd80 | cd80 antigen | -1,6 |
|  | Il6 | interleukin 6 | -1,7 |
|  | Il2ra | interleukin 2 receptor, alpha chain | -1,7 |
|  | Nos2 | nitric oxide synthase 2 | -1.8 |
|  | Socs1 | suppressor of cytokine signaling 1 | -1,9 |
|  | Cxcl10 | chemokine ligand 10 | -1.9 |
|  | Ptgs2 | prostaglandin-endoperoxide synthase 2 | -2,1 |
|  | Cxcl11 | chemokine ligand 11 | -3,0 |
|  | Ifng | interferon gamma | -3,2 |
