# Supplementary material for: A Strong Anti-Inflammatory Signature Revealed by Liver Transcription Profiling of Tmprss6−/− Mice
Source: PLoS One. 2013 Jul 29;8(7):e69694. doi: 10.1371/journal.pone.0069694 (PMC3726786; doi:10.1371/journal.pone.0069694)
Supplement: Table S3 — Comparison between Tmprss6 KO and Hfe −/− on the expression of selected liver genes. (DOCX) [file pone.0069694.s009.docx]

Table S3. Comparison between *Tmprss6* KO and Hfe-/-

|  | **Genes** | **Description** | **Tmprss6 KO vs IDA**  **(high Hamp)^b^** |
| --- | --- | --- | --- |
| **Down-regulated in**  **Hfe^-/-^ vs IL**  **(low Hamp)^a^** | Hamp1 | hepcidin | ↑ |
|  | Hbb-b1 | Hemoglobin, beta chain | ↑ |
|  | H2-Ab1 | Histocompatibility2, class II antigen A, b | ↑ |
|  | H2-Eb1 | Histocompatibility2, class II antigen E, b | ↑ |
|  | Egr1 | Early growth response | ≈ |
|  | Gsta2 | Glutathione S transferase, alpha 2 | ≈ |
|  | H2-Aa | Histocompatibility2, class II antigen A, a | ≈ |
|  | Cd74 | Polypeptide of MHC II | ≈ |
|  | Cyp4a14 | Cytochrome P450, family 4a14 | ≈ |
|  | Cish | Suppressor of cytokine signaling | ↓ |
|  | Phlda1 | Pleckstrin like domain, family A1 | ↓ |
|  | Socs3 | Suppressor of cytokine signaling 3 | ↓ |
|  | Cyp26b1 | Cytochrome P450, family 26b1 | ↓ |
|  | Rnf186 | Ring finger protein 186 | not found |
| **Up-regulated**  **in**  **Hfe^-/-^ vs IL**  **(low Hamp)^a^** | Il6ra | Interleukine 6 receptor, a | ↓ |
|  | Hp | haptoglobin | ≈ |
|  | Mup4 | Major urinary protein 4 | ≈ |
|  | Saa1 | Serum amyloid A1 | ≈ |
|  | Lpin1 | Lipin 1 | ≈ |
|  | Dct | Dopachrome tautomerase | ≈ |
|  | Saa2 | Serum amyloid A2 | ≈ |
|  | Saa3 | Serum amyloid A3 | ≈ |
|  | Angptl4 | Angiopoietin-like 4 | ≈ |
|  | Dio1 | Deiodinase, iodothyronine, type I | ≈ |
|  | Ppp1r3b | Protein phosphatase 1, subunit 3B | ≈ |
|  | Serpina12 | Serine peptidase inhibitor A12 | ≈ |

a: modified from Rodriguez et al., PLoSONE 2009, 4 (9):e7212.

b: data obtained from total liver gene expression profiling of Tmprss6^-/-^ mice compared to iron deficient control littermates

↓: genes down-regulated in Tmprss6 vs ID

↑: genes up-regulated in Tmprss6 vs ID

≈: genes not changed between Tmprss6 and ID
