# Supplementary material for: A Strong Anti-Inflammatory Signature Revealed by Liver Transcription Profiling of Tmprss6−/− Mice
Source: PLoS One. 2013 Jul 29;8(7):e69694. doi: 10.1371/journal.pone.0069694 (PMC3726786; doi:10.1371/journal.pone.0069694)
Supplement: Table S4 — List of oligonucleotides primers used for qRT-PCR. (DOCX) [file pone.0069694.s010.docx]

Table S4. Oligonucleotides Primers (Applied Biosystem, Carlsbad, CA, USA) used for qRT-PCR by TaqMan.

| Name | Id |
| --- | --- |
| Apoa4 | Mm00431814_m1 |
| Atf3 | Mm00476032_m1 |
| C9 | Mm00442739_m1 |
| Ccnd1 | Mm00432359_m1 |
| Cd40 | Mm00441891_m1 |
| Cxcl1 | Mm04207460_m1 |
| Cyp2b9 | Mm00657910_m1 |
| Efna1 | Mm01212795_m1 |
| Fbxo21 | Mm01208074_m1 |
| Gdf15 | Mm00442228_m1 |
| Hamp | Mm00519025_m1 |
| Icam1 | Mm00516023_m1 |
| Il1rn | Mm00446186_m1 |
| Irak3 | Mm00518541_m1 |
| Jun | Mm00495062_m1 |
| Myd88 | Mm00440338_m1 |
| Nfkbiz | Mm00600522_m1 |
| Slc2a2 | Mm00446229_m1 |
| Socs3 | Mm00545913_m1 |
| Tlr2 | Mm00442346_m1 |
| Tnfaip2 | Mm00447578_m1 |
